# Sustainable shrimp farming in Sri Lanka; Utilization of BMPs and antibiotics use

**DOI:** 10.5455/javar.2024.k744

**Published:** 2024-03-12

**Authors:** Hiroichi Kono, Takahiro Sajiki, M. N. D. F. Abeykoon, Keisuke Kato, Tiana N. Randrianantoandro

**Affiliations:** 1 Department of Agro-Environmental Science, Obihiro University of Agriculture and Veterinary Medicine, Obihiro, Japan; 2 Japan Fisheries Research and Education Agency, Fisheries Technology Institute, Yokohama, Japan; 3 Department of Agro-Management, Antananarivo University, Ecole Supérieure des Sciences Agronomiques, Antananarivo, Madagascar

**Keywords:** Antibiotics, best management practices, shrimp farming, Sri Lanka

## Abstract

**Objective:**

In the context of the fast-expanding shrimp farming industry in Sri Lanka, this study aimed to evaluate the distribution and understanding of BMPs, examine the relationship between BMPs and disease outbreaks, and analyze the current antibiotic usage trends through a fact-finding survey.

**Materials and Methods:**

A questionnaire survey was conducted in 131 shrimp farms located in Puttalam District in North Western Province, where shrimp farming is thriving in Sri Lanka. The survey was conducted from September to October 2021. In addition, in August 2022 and August 2023, interviews were conducted with shrimp farms in Puttalam district and Batticaloa district, shrimp hatcheries, and export companies. Data were analyzed using descriptive statistics, Probit, and Tobit regression analysis.

**Results:**

The item-count technique revealed a significant (p < 0.05) difference in the use of antibiotics without consulting experts, signifying inappropriate use. No aquaculture farmer reported being unaware of BMPs, and among the 45 farmers who attended a BMPs seminar, 30 claimed to possess a good understanding of BMPs. Probit and Tobit regression results revealed that the rate of understanding of BMPs, education level, and obtaining information on the sanitation management of shrimp farming from extension and guidance organizations were inversely associated with both disease incidence in shrimp farming and shrimp discards.

**Conclusion:**

Although the use of antibiotics is prohibited in BMPs, the analysis results suggest inappropriate use of antibiotics. The findings indicate that enhanced BMP understanding can reduce disease incidence and shrimp discards, emphasizing the need for incentives to promote BMP adoption and reduce the necessity for antibiotics.

## Introduction

Shrimp has emerged as an integral part of global aquaculture and the world food market [1]. It is among the most traded seafood products in the international market and plays a vital role for developing countries, notably in Asian countries that contribute to approximately 80% of the world’s shrimp exports [2]. In these developing countries, semi-intensive and intensive shrimp farming serves as a lucrative venture and a major industry for earning foreign currencies [3].

Disease outbreaks in shrimp farming directly influence the global shrimp supply, prices, and consequently, overall profitability [4]. Hence, disease control is vital in shrimp farming. In the early 2000s, shrimp farming in Thailand suffered a severe setback due to the spread of diseases such as yellowhead disease [5]. Similarly, in China, the shrimp industry collapsed due to the white spot syndrome between 1992 and 2000 [6]. In 1995, FAO and the Network of Aquaculture Centers in Asia devised a hygiene-management movement called Better Management Practices (BMPs) [7,8]. Under BMPs, the use of antibiotics in shrimp farming is prohibited. This is for public health reasons as drug-resistant bacteria emerge that are resistant to excessive use of antibiotics, threatening human health. However, in many developing countries, inappropriate use of antibiotics has been reported, despite a ban on their use [9,10]. Consequently, the quarantine standards for shrimp imported from developing countries have been tightened [11].

Shrimp farming in Sri Lanka accounts for half of the total export earnings in the aquaculture sector [12]. It has gained significance in recent years, achieving the second-highest foreign exchange earnings among export fisheries in 2021 [13]. Black tiger shrimp were popular in Sri Lanka until 2018. However, due to diseases and other factors, the production volume for black tigers has persistently fluctuated. Note 1: The spread of yellowhead disease and white spot disease has had a major impact on shrimp exports from Sri Lanka [14]. The Sri Lankan government introduced Vannamei shrimp in 2018. The farming of the species has progressed rapidly, and the annual production of Vannamei, which was 543MT in 2019, had rapidly expanded to 10,535MT by 2021, surpassing the production of the black tiger (3,880MT). Note 2: Data source: NAQDA. One of the primary factors driving the rapid expansion of Vannamei farming is that it has been clarified that it is highly resistant to diseases and can be cultivated in overcrowded conditions, resulting in high economic returns [15].

BMPs were developed for both agriculture and aquaculture as a means for farmers to improve economic gains while reducing negative impacts on the environment. BMPs for shrimp culture are based on the principles of sustainable shrimp farming developed by consortia of international agencies, including the World Wide Fund for Nature, and the code of conduct for responsible fisheries prepared by FAO [8]. The international principles and code have been adapted to national levels in each shrimp-farming country. In the case of Sri Lanka, 13 guidelines for BMPs were developed by the National Aquaculture Development Authority (NAQDA). Farmers can only obtain shrimp-farming licenses after agreeing to comply with BMPs. As of 2018, approximately 700 farms in Sri Lanka had obtained shrimp-farming licenses from NAQDA [16]. Dissemination and enlightenment training on BMPs is regularly provided to farmers’ organizations by the NAQDA.

In Sri Lanka, the use of antibiotics is prohibited under BMPs, and NAQDA regularly monitors their use. If a regular NAQDA inspection confirms the use of antibiotics, the shrimp farming license will be revoked. However, in Sri Lanka, although three types of antibiotics (Chloramphenicol, Nitrofurans, and Malachite green) are prohibited, the use of other antibiotics is not [12]. That is, the use of antibiotics is prohibited under BMPs, and NAQDA states that there is no use of antibiotics in shrimp farming. Nevertheless, the use of antibiotics on shrimp-farming production sites is suspected, and the actual situation remains unknown. Conversely, in response to the 2020 economic crisis, Sri Lanka is focusing on enhancing its export-earning sectors, particularly the shrimp industry. To achieve this, adhering to Best Management Practices (BMPs) is essential for promoting sustainability and expanding shrimp exports.

Within this context, the purpose of this study is to clarify the status of the spread and understanding of BMPs in shrimp farming, the relationship between BMPs and disease outbreaks, and the actual situation of antibiotic use based on a fact-finding survey conducted in Sri Lanka, where shrimp farming is progressing rapidly.

## Materials and Methods

### Survey method

From September to October 2021, we conducted a questionnaire survey of 131 shrimp farms in Puttalam District, North Western Province, where shrimp farming is thriving in Sri Lanka. In the questionnaire, we collected information on sales performance, including shrimp sanitary management methods, shrimp-waste amount, use of antibiotics, understanding of BMPs, and farmers’ characteristics on shrimp farms. In addition, in August 2022 and August 2023, interviews were conducted with shrimp farms in Puttalam district, Batticaloa district in the east, shrimp hatcheries, and export companies.

### Analysis method

We assessed the status of participation in the BMPs training conducted by NAQDA and the degree of understanding of BMPs using a questionnaire. BMP comprehension was evaluated on a 4-point scale from “I do not know” to “I know BMPs very well.” In addition, the degree of implementation of each item of BMPs was indexed and analyzed.

Next, the status of disease occurrence in shrimp farming and the amount of shrimp discarded due to disease were used as dependent variables, and the degree of understanding of BMPs and farmers’ characteristics were used as explanatory variables. Probit regression analysis, which is a binary choice model, and Tobit regression analysis, which is a truncated regression model [17], were conducted to examine the relationship between the two. Both models are used when the data range for the dependent variable is limited. The dependent variable in the probit model is a dummy variable that assumes 1 when there is a disease outbreak and 0 otherwise. The dependent variable in the Tobit model is the amount of shrimp discarded per unit area if there were shrimp discarded due to disease in the previous year; it equals zero if there were no discards.

Under BMPs, shrimp farmers in Sri Lanka are prohibited from using antibiotics. However, in local interviews and related studies, it has been suggested that shrimp farms use antibiotics [18]. Asking aquaculture farmers about their use of banned antibiotics is extremely sensitive, and one cannot expect an honest answer.

In this study, the item-count technique (ICT) is used to estimate the percentage of aquaculture farmers who inappropriately use antibiotics. ICT is one of the survey methods that indirectly asks and confirms content that cannot be expected to be answered honestly, such as socially undesirable content (for example, the use of drugs) in survey items [19].

In the Sri Lanka survey, we prepared the following two types of sensitive items: (a) “I do not know exactly the efficacy, usage, dosage, and withdrawal period of antibiotics used for shrimp.” (b) “I sometimes use antibiotics without consulting a professional (such as a veterinarian at a government guidance agency).” In the actual survey, three types of questionnaire forms were used. Questionnaire 1 is an indirect one that includes multiple nonsensitive items in addition to a sensitive item (a). Questionnaire 2 is an indirect one, comprising a sensitive item (b) and other nonsensitive items. Questionnaire 3 directly asks about two sensitive items (a and b). For each item, the respondents were asked to only provide the number of questions that they felt applied to them. That is, they did not directly respond to each question.

Below are the five indirectly asked confirmation items in Survey Table 1. The sensitive item is ④, and the others are nonsensitive items. Answer only with a number that corresponds to the count of the number of items that apply to you among the following:

① I almost always purchase shrimp food from the same store.

② I regularly check the health condition of my shrimp.

③ I have friends who can consult with me about shrimp diseases.

④ I do not know exactly the efficacy, usage, dosage, and prohibition period of pharmaceuticals used for shrimp.

⑤ I have had my farmed shrimp stolen before.

The percentages of affirmative responses to the items in Survey Tables 1–3 are represented by *α*, *β*, and *γ*, respectively. If *α* > *γ* and *β* > *γ*, inappropriate use of antibiotics is suspected. (Note 3: The numbers of farm households in the three types of survey tables used in the Puttalam area are 50, 50, and 30, respectively, for a total of 130 households).

## Results

Table 1 summarizes the BMP training provided by NAQDA and the level of BMP comprehension. No aquaculture farmers answered that they did not know about BMPs, and 30 of the 45 aquaculture farmers who had attended a BMPs seminar answered that they had a good knowledge of BMPs. In addition, 19 aquaculture farmers answered that they “know very well,” even if they had never participated in a BMP seminar.

**Table 1.** Relationship between understanding of BMP and BMP seminars.

Participation in BMP	Rate of understanding of BMP		
Seminar	Understand a little	Understand	Understand it well
No	33 (25.4)	33 (25.4)	19 (14.6)
Yes	0 (0.0)	15 (11.5)	30 (23.1)
Total	33 (25.4)	48 (36.9)	49 (37.7)

Note: No. of aquaculture farming households that fall under each column. The numbers in parentheses represent the proportions (%) in all farming households.

**Table 2.** Definition of variables used for analysis and descriptive statistics.

Variable name	Definition	Mean	Std. dev.	Mini	Maxi
Shrimp disease outbreak	Shrimp disease outbreaks in the last year (Yes = 1, No = 0)	0.351	0.47	0	1
Shrimp discard amounts	Discarded shrimp per unit farm area *n* the past 1 year	0.116	0.18	0	0.65
Age	7-point scale, where 1 = 15 years old, 2 = 25 years old, up to 6 = 65, 7 = 75.	3.824	0.94	2	7
Education level	4-point scale, where None = 1, up to 4 = high school graduate	3.099	1.00	2	4
Farm area	Logarithm of shrimp farming area	9.613	0.60	8.30	10.94
Neighboring disease dummy	Neighboring disease outbreaks (Yes = 1, No = 0)	0.351	0.49	0	1
Information source dummy	Information sources for shrimp farming techniques (Government Guidance Organization = 1, Others = 0)	0.415	0.83	0	1
Rate of understanding of BMP	4-point scale, do not understand = 1 up to understand well = 4	3.099	0.83	2	4

**Table 3.** Analysis of factors in the incidence of disease and amount of shrimp discarded.

Explanatory variable	Probit model			Tobit Model		
	Dependent variable: shrimp disease outbreak			Dependent variable: shrimp discard amounts		
	Coefficient	Standard error		Coefficient	Standard error	
Constant terms	−2.192	2.484		−0.331	0.678	
Rate of understanding of BMP	−0.513	0.191	***	−0.116	0.052	**
Age	−0.257	0.188		−0.089	0.045	**
Education level	−0.359	0.199	*	−0.132	0.050	***
Farm area	0.480	0.242	**	0.119	0.068	*
Neighboring disease dummy	2.356	0.313	***	0.593	0.067	***
Information source dummy	−0.560	0.317	*	−0.274	0.079	***
log-likelihood	−44.047			−39.823		

Note: *n* = 130; *,**, and *** indicate statistical significance at 10%, 5%, and 1%, respectively.

Table 2 shows the descriptive statistics for the variables used in the Probit and Tobit models, and Table 3 shows the measurement results.

The analysis results (Table 3) show almost the same tendency for the probit and Tobit models. First, an increased understanding of BMPs tends to reduce the incidence of disease in shrimp farming and the amount of shrimp discarded. In the past year, 46 farms, or 35% of the surveyed households, reported disease outbreaks in shrimp farming. Second, it was found that when the level of education was high and information on the sanitation management of shrimp farming was obtained from extension and guidance organizations, the incidence of disease decreased and the amount of shrimp discarded tended to decrease. Third, it became clear that the larger the aquaculture area and the greater the number of disease outbreaks in neighboring farms, the greater the number of disease outbreaks and the greater the amount of shrimp discarded.

Table 4 presents the results of dividing aquaculture farmers into two groups according to their level of understanding of BMPs (as in Table 2) and statistically testing the difference between the two groups in the sanitation-management action index specified by BMPs. From the indicators for each hygiene-management behavior of Group (A) who answered “well understand” and “know BMPs,” it was confirmed that there was a statistically significant difference in many items compared to the index. It was also revealed that there was a statistically significant difference between the A and B groups in terms of the total index value.

Table 5 shows the ICT analysis results. We begin our explanation with the first item, “I do not know exactly the efficacy, usage, dosage, and withdrawal period of antibiotics used for shrimp.” Based on the analysis, although statistically significant, the percentage of responses to direct questions (60.0%) was higher than that of those to indirect questions (26.0%). This indicates that the sensitive item was not a sensitive question for aquaculture farmers, a result contrary to our expectations. In truth, the farmers would not be legally punished for ignorance about sensitive items and were not in danger of losing their shrimp farming permits.

Consequently, it is considered that this is not a sensitive item for aquaculture farmers.

Next, the second item, “I sometimes use antibiotics without consulting experts (veterinarians of government guidance agencies, etc.),” is explained. In the direct questionnaire, all the respondents said that they would never use antibiotics without veterinary approval. However, in the indirect question, 18% of the farmers said that they sometimes used antibiotics without a veterinarian’s permission. The binomial test shows this difference to be statistically significant at the 5% level. It became clear that the item “sometimes use antibiotics without expert permission” was a sensitive and difficult question for aquaculture farmers. In other words, the results indicate the possibility of inappropriate use of antibiotics in shrimp farming in Sri Lanka.

## Discussion

The number of aquaculture farmers who have participated in the BMPs course is only 45, which may appear to be a small number. However, only the representatives of each district participate in the seminars, and after the seminars are over, the contents of the seminars are communicated to other aquaculture farmers. NAQDA also distributes leaflets on BMPs to all aquaculture farmers, and therefore, it is believed that even farmers who have not participated in the seminars have gained a certain degree of understanding of BMPs. The analysis revealed that an increased understanding of BMPs has contributed to lowering disease incidence and reducing shrimp waste in shrimp farming in the study area. This is because the items stipulated in BMPs, such as “stocking density” and “feed amount,” as shown in Table 4, are being properly observed, and this has led to a decrease in the incidence of diseases in shrimp farming and a reduction in the amount of shrimp discarded. This research finding is consistent with similar studies conducted in many other developing countries affirming that BMP adoption reduces the possibility of disease prevalence [20–22].

**Table 4.** Statistical tests for differences in BMP hygiene behavior indicators.

Hygenic efforts	A (*n =* 96)		B (*n =* 34)		
(1) Using water and bottom sediment improvers during shrimp farming [Min0, Max1]	0.979	(0.143)	0.794	(0.410)	**
(2) Report to government guidance agency when shrimp is sick [Min1, Max3]	2.100	(0.784)	1.620	(0.697)	***
(3) Cleaning the farm before shrimp farming [Min0, Max1]	0.969	(0.174)	0.971	(0.174)	
(4) Observation frequency of water quality and bottom sediment during shrimp farming [Min1, Max4]	3.010	(0.421)	2.941	(0.547)	
(5) Using probiotics for shrimp farming [Min1, Max3]	2.969	(0.226)	3.000	(0.000)	
(6) Treatment of aquaculture pond wastewater with disinfectant [Min1, Max3]	1.381	0.622	1.735	(0.864)	*
(7) Observation frequency of shrimp health condition [Min1, Max5]	2.990	1.015	3.735	(0.751)	***
(8) Shrimp rearing density is appropriate [Min1, Max3]	1.371	0.618	1.206	(0.538)	**
(9) Appropriate feeding amount of shrimp [Min1, Max3]	1.557	0.790	1.265	(0.666)	**
(10) Do not abandon diseased shrimp in the vicinity of farms or into rivers [Min1, Max3]	1.742	0.916	1.059	(0.343)	***
total	22.598	(3.530)	20.061	(1.540)	***

Note 1: Each index is evaluated using the maximum and minimum values in parentheses [ ], and the average value is shown. The figures in parentheses ( ) are standard deviations.

Note 2: *,**, and *** represent statistical significance at 10%, 5%, and 1%, respectively.

Note 3: A represents the group “I know BMP well” and “I know BMP,” and B represents the group “I know BMP a little”.

**Table 5.** ICT results.

Confirmed sensitive questions	Indirect question (%)	Direct question (%)	Binomial test	
(1) I do not exactly know the efficacy, usage, dosage, and period of prohibition of use of antibacterial agents used for shrimp (*n =* 50)	*α* = 26.0	γ 1 = 60.0	0.015	**
(2) I sometime use antibiotics without the permission of experts (veterinarians, etc. of guidance institutions) (*n =* 50)	*β* = 18.0	γ 2 = 0.0	0.039	**

Note: ** represents statistical significance at 5%; direct-question sample size *n =* 30.

Shrimp farming in Sri Lanka requires a permit from NAQDA, which requires compliance with BMP regulations and prohibits the use of antibiotics. All farms surveyed are registered with NAQDA and licensed for shrimp farming. Shrimp farmers should be complying with the BMP regulations; however, the ICT results indicated otherwise. Shrimp farmers may be using antibiotics without professional approval. These results are consistent with a study [4] that shows the detection of antibiotics in shrimp sampled from farms in Sri Lanka and interviews with relevant local institutions. Previous studies have also emphasized the issue of information asymmetry in farmers’ antibiotic usage within the context of antibiotic governance [23,24].

Among the reasons for the asymmetry of information is that the guidance agency (principal) cannot completely observe the behavior of the shrimp farmer (agent) [25]. Sharing symmetrical information on antibiotic use can be achieved through effective communication between farmers and government authorities [26]. However, farmers’ behavior toward antibiotic use is influenced by many factors, including government regulation, the cost-effectiveness of antibiotic usage, inelastic demand, overestimations of disease-related losses, biased trust in the efficacy of veterinary antibiotics, and a heavy dependence on antibiotics [23].

Although the use of antibiotics is prohibited in BMPs, the analysis results suggest inappropriate use of antibiotics. However, in Sri Lanka, only one case of the revocation of a shrimp-farming permit has been reported thus far, and this was not for inappropriate use of antibiotics but for failure to comply with some BMPs clauses (Note 4: From a local interview).

According to the principal-agent theory, to alleviate the problem of information asymmetry, when a shrimp farmer engages in a desired behavior (not using antibiotics) under BMPs, it is important to provide an incentive such that their gain (*Y*) is higher than the gain (*X*) from undesirable behavior (using antibiotics) so that the incentive-compatibility condition is established (*Y* > *X*) [10]. As demonstrated in this study, a better understanding of BMPs will reduce the incidence of disease in shrimp farming and reduce the amount of shrimp discarded. The effect of BMP adherence on shrimp farming (gain: *Y*) has not been clarified in previous studies. It is necessary to widely disseminate this point to shrimp farms and related organizations to provide incentives for BMP implementation and reduce the necessity of antibiotic use. It is also important to directly obtain samples from shrimp farms and conduct antibiotic tests. Based on the results, regulations regarding shrimp farming licenses should be tightened. In addition, institutional reforms that promote effective penalties (negative payoff: *X*) when the use of antibiotics is found will be necessary to establish incentive-compatibility conditions.

One of the limitations of this study was the utilization of an indirect approach (ICT) to analyze antibiotic usage among shrimp farmers. This methodology was adopted due to farmers’ hidden behavior regarding the use of antibiotics in Sri Lanka.

## Conclusion

There is an information asymmetry between guidance agencies such as NAQDA and shrimp farmers regarding the use of antibiotics. It should be pointed out that shrimp farmers have hidden behaviors regarding the use of antibiotics, while guidance agencies (such as NAQDA) may not be able to observe such problems. Although the use of antibiotics is prohibited in BMPs, the analysis results suggest inappropriate use of antibiotics. As demonstrated in this study, a better understanding of BMPs will reduce the incidence of disease in shrimp farming and reduce the amount of shrimp discarded. It is necessary to widely disseminate this point to shrimp farms and related organizations to provide incentives for BMP implementation and reduce the necessity of antibiotic use. Based on the results, regulations regarding shrimp farming licenses should be tightened. In addition, institutional reforms that promote effective penalties when the use of antibiotics is found will be necessary to establish incentive-compatibility conditions. Shrimp exports are a promising means of earning foreign currency for Sri Lanka, which is looking for opportunities to earn foreign currency due to the economic crisis. The development of sustainable shrimp farming in compliance with BMPs is expected in Sri Lanka.

## List of abbreviations

BMPs, Best management practices; FAO, Food and Agriculture Organization of the United Nations; ICT, item-count technique; NAQDA, National Aquaculture Development Authority.
